# Use and Quality of Blood Cultures for the Diagnosis of Bloodstream Infections: A Cross-Sectional Study in the Ho Teaching Hospital, Ghana, 2019–2021

**DOI:** 10.3390/ijerph20176631

**Published:** 2023-08-23

**Authors:** Emily Boakye-Yiadom, Robinah Najjemba, Pruthu Thekkur, Appiah-Korang Labi, Julita Gil-Cuesta, Karikari Asafo-Adjei, Prosper Mensah, Elburg van Boetzelaer, Nasreen S. Jessani, Verner Ndudri Orish

**Affiliations:** 1 Department of Microbiology and Immunology, University of Health and Allied Sciences, Ho PMB 31, Volta Region, Ghana; vorish@uhas.edu.gh; 2 Laboratory Department, Ho Teaching Hospital, Ho P.O. Box MA 374, Volta Region, Ghana; kk.asafoadjei@gmail.com (K.A.-A.); prosmens@yahoo.co.uk (P.M.); 3 Independent Public Health Consultant, 1202 Geneva, Switzerland; robinahnajjemba@yahoo.co.uk; 4 International Union Against Tuberculosis and Lung Disease, 68 Boulevard Saint Michel, 75006 Paris, France; pruthu.tk@theunion.org; 5 Ghana Country Office, World Health Organization, 7 Ameda Street, Roman Ridge, Accra P.O. Box MB 142, Ghana; labia@who.int; 6 Luxembourg Operational Research Unit, Operational Centre Brussels, Médecins Sans Frontières, Rue Arbre Benit 46, 1050 Brussels, Belgium; giljulita@gmail.com; 7 Luxembourg Operational Research Unit, Médecins Sans Frontières, 68 Rue de Gasperich, L-1617 Luxembourg, Luxembourg; elburgvb@gmail.com; 8 Centre for Evidence-Based Health Care, Division of Epidemiology and Biostatistics, Department of Global Health, Stellenbosch University, Tygerburg 7505, South Africa; njessani@sun.ac.za; 9 Department of International Health, Johns Hopkins Bloomberg School of Public Health, 615 North Wolfe Street, Baltimore, MD 21205, USA; 10 Sickle Cell Disease Unit, Department of Internal Medicine, Ho Teaching Hospital, Ho P.O. Box MA 374, Volta Region, Ghana

**Keywords:** blood culture, bloodstream infection, antimicrobial resistance, antimicrobial susceptibility testing, laboratory quality, sepsis, neonatal sepsis, Ghana, operational research, SORT IT

## Abstract

Blood Culture and Drug Susceptibility Testing (CDST) remains vital for the diagnosis and management of bloodstream infections (BSIs). While the Ghana National Standard Treatment Guidelines require CDST to be performed in each case of suspected or clinically diagnosed BSI, these are poorly adhered to in the Ho Teaching Hospital (HTH). This study used secondary medical and laboratory records to describe blood CDST requests by clinicians and the quality of CDST processes for the diagnosis of BSI among patients admitted to HTH from 2019 to 2021. Of 4278 patients, 33% were infants. Pneumonia and neonatal sepsis cases were 40% and 22%, respectively. Only 8% (351/4278) had blood CDST requested. Of 94% (329/351) blood CDST processed and reported, only 7% (22/329) were culture-positive, with likely contaminants being recovered from 16% (52/329) of the specimens. The duration from admission to request was 2 days (IQR: 0–5), and Further qualitative studies must be conducted to understand the reasons for low blood CDST utilisation among clinicians and the patient outcomes. Targeted interventions are required to enhance the utilisation of blood CDST by clinicians and the quality of laboratory processes.

## 1. Introduction

Bloodstream infections (BSIs) are associated with severe morbidity and mortality and affected patients often require inpatient care, extensive investigations and prolonged antibiotic use [1]. Prompt and effective management helps to prevent adverse outcomes such as septic shock, major organ failure, or death [1,2].

BSI is classified as primary when infections occur de novo or secondary as a result of focal infections such as meningitis, osteomyelitis and pneumonia [1,3]. A review of the burden of BSI in Europe and North America estimated a combined annual incidence of about 2 million and 250,000 deaths due to sepsis [4]. The burden of BSI is complicated by a global rise in antimicrobial resistance (AMR), with a major impact expected in low- and middle-income countries (LMICs) [5,6,7,8,9]. In West Africa, several studies have reported a high prevalence of AMR among hospitalised patients with BSI [10,11,12]. Rising AMR in LMICs is compounded by poor access to diagnostic microbiology services, which consequently affects the quality of healthcare delivery [13,14].

Laboratory identification of the pathogen and its antimicrobial susceptibility are essential to guide appropriate targeted antimicrobial therapy for severe bacterial infections, including BSI [2,15,16,17]. Despite the general shift toward the utilisation of rapid diagnostic tests, including molecular tests, ‘traditional’ phenotypic tests like Gram stain with microscopy and Culture and Drug Susceptibility Testing (CDST) remain integral for the diagnosis and management of BSI [18,19,20,21]. Blood CDST, when performed in a timely manner and coupled with rapid communication of results, effectively improves clinical outcomes of patients with BSI [19,22]. However, the clinical utility of blood CDST can be affected by factors such as the timing of sample collection, sample volume, contamination rate, time-to-detection of growth, and turnaround time (TAT) for laboratory processing [22]. These factors, all components of a laboratory quality system, influence the accuracy, reliability and timeliness of blood CDST results and, consequently, physician trust and, therefore, use of the test [23]. Periodic assessments of access to and quality of blood CDST services are therefore important for identifying and addressing systemic issues affecting blood CDST utilisation, improving laboratory efficiency and enhancing clinical practice [18,24,25].

The seventh edition of the Ghana National Standard Treatment Guidelines requires blood CDST to be performed in each case of suspected or clinically diagnosed BSI [26]. However, two studies conducted at the Ho Teaching Hospital (HTH) in the Volta Region of Ghana between 2019 and 2020 established that 95% of the antimicrobial therapy administered to inpatients with conditions like pneumonia and sepsis was empirical, thereby implying poor adherence to the guidelines [27,28].

In this study, we described trends in blood CDST requests and the quality of blood CDST laboratory services for the diagnosis of BSI among patients admitted to HTH from 2019 to 2021. Identifying the patterns of laboratory utilisation in BSI and the quality of blood CDST performed in these instances helped to identify areas for strengthening antimicrobial stewardship in our facility.

## 2. Materials and Methods

### 2.1. Study Design

This was a hospital-based cross-sectional study using secondary data from routine medical and laboratory records.

### 2.2. General Setting

Ghana is an LMIC of 30.8 million persons [29] located in West Africa [30]. Healthcare is provided by both public and private facilities. Public healthcare facilities, including those owned by faith-based organisations, are organised into primary, secondary and tertiary levels and are managed by the Ministry of Health (MoH). The distribution of these facilities and skilled healthcare staff disproportionately favours the two largest cities, Accra and Kumasi [31,32]. Pneumonia, sepsis and septicaemia were among the top 10 causes of hospital admissions in 2018 [32]. Blood CDST services are mostly available in tertiary public health facilities and a few private medical laboratories. The National Health Insurance Scheme (NHIS) provides healthcare financing for about 50% of the population, and it covers the cost of diagnostic tests [33]. The national AMR surveillance project was initiated in 2018, and it comprises 11 laboratories reporting from public human and animal health facilities located in five regions, including the Volta region [34], as one of 16 administrative regions, accounting for 5.4% of the total population. The hospital admission rate within the region is 56.9/1000 population, with an average hospital stay of 4.5 days [29].

### 2.3. Specific Setting

HTH is a 340-bed tertiary facility affiliated with the University of Health and Allied Sciences (UHAS) and is located in Ho, the capital of the Volta region. It provides specialist outpatient and inpatient services to the Volta and Oti regions, neighbouring parts of the Eastern region of Ghana and the Republic of Togo. HTH has a well-resourced Laboratory Department, which is the main laboratory equipped to conduct bacterial cultures in the Volta and Oti regions. The NHIS paid for almost 90% of patients accessing care in the hospital in 2021 [35]. During the study period, the total cost of blood CDST was approximately seven US dollars for persons who did not subscribe to the NHIS. Inpatients are billed upon discharge.

The hospital employs an electronic health records (EHR) system for all its activities. Until November 2020, this was performed via the Health Administration and Management (HAMS) software, which was supplemented by paper folders for inpatient clinical notes. Since December 2020, this has been replaced by the Lightwave Health information management system (LHIMS) as part of the roll-out of a National E-Health project [36]. The LHIMS is web-based and contains patient information and clinical notes, laboratory records, results of diagnostic tests, and pharmacy records, all of which feed into a centralised data repository. The system creates a unique user identification number (hospital ID) for each patient at the first visit, and this is permanently retained for subsequent visits. A total of 30,512 admissions across all wards and 1130 blood CDST were recorded during the study period.

#### Workflow for Blood CDST Processing in HTH

An attending clinician suspecting a BSI requests for a patient’s blood CDST through the EHR. Blood culture bottles are obtained from the laboratory by healthcare personnel, and sampling is performed by a nurse or clinician following strict aseptic techniques. Ideally, the request and sample collection should be performed within hours of recognition of a BSI and prior to the initiation of empirical antimicrobial therapy [2,19]. The recommended blood culture sample to draw per bottle is 1–3 mL for children and 8–10 mL for adults as per standard protocol [1,37]. Although international guidelines recommend sampling for a blood culture request to be two or more sets of two samples, each taken within a 24 h period, the practice in our facility, as in other LMICs, has been to use unitary samples per request [17,19,38,39].

The samples are immediately incubated for seven days upon receipt at the laboratory, which is expected to occur at most four hours after sample collection [19,40]. From January 2019 to September 2021, a manual incubator was used. Consequently, incubated samples were manually inspected for signs of microbial growth at least twice daily. However, from October to December 2021, this was replaced with a BACTEC™ incubator (Becton Dickinson), which additionally performs automated continuous scanning of incubated samples to detect microbial growth. Regardless of the incubator used, phenotypic tests are used to identify the specific pathogens for all positive cultures as per standard protocol [39]. This includes Gram staining with microscopy and sub-culturing on appropriate media. Specific panels of biochemical tests are then applied to identify the isolated bacteria. Blind sub-cultures are performed on the second and seventh days for samples with no signs of growth after manual incubation [39,40]. Antimicrobial susceptibility testing by disk diffusion is then performed according to the pertaining guidelines of the Clinical and Laboratory Standards Institute (CLSI) [41].

The results of all tests conducted as part of blood CDST processing are logged in a sample register. Preliminary reports for all blood CDST are expected within 72 h of incubation [17,40]. However, this practice is not routine in our facility. The final test result is issued via the EHR after antimicrobial susceptibility testing. Available literature increasingly supports direct communication of preliminary and final test results, especially of critical values like the Gram staining result, the identity of the organism and the result of antimicrobial susceptibility testing by telephone to the attending physician as soon as they are available so that appropriate changes in therapy can be initiated [17,19,22,40]. However, this practice is not routine in our facility. Based on the phenotypic system of blood CDST processing in place at HTH, final reports are expected to be issued within 5–7 days from sample receipt [17,40].

### 2.4. Study Population

This included all inpatients admitted to HTH from January 2019 to December 2021 with at least one clinical diagnosis or suspicion of a BSI. The study population comprised 4278 inpatients with BSIs.

### 2.5. Definition of Terms

BSI: clinical diagnosis or suspicion of at least one primary or secondary infection of the bloodstream suspected or proven to be of bacterial or fungal origin [1,3,6,17,40,42]. Parasitic and viral bloodstream infections are, therefore, excluded.

Primary BSI: sepsis, septicaemia, severe sepsis, septic shock, neonatal sepsis, sepsis of newborn, endocarditis, catheter-related BSI or central line-associated BSI.

Secondary BSI: puerperal sepsis, septic abortion, meningitis, intracerebral abscess, pyelonephritis, enteric or typhoid fever, typhoid enteritis, typhoid perforation, pneumonia, bronchopneumonia, aspiration pneumonia, osteomyelitis, septic arthritis, intra-abdominal abscess, pelvic abscess, spontaneous bacterial peritonitis, peritonitis, cholecystitis, cholangitis, biliary tract infection or fever of unknown origin.

Presumed BSI: patients without documentation of a specific infection but for whom a blood CDST was requested, implying a clinical suspicion of a BSI [1,17,40,43].

Yield (diagnostic yield): proportion of true bacteraemia detected by blood CDST. Represents organisms already present in the patient’s bloodstream during sampling.

Contamination: proportion of false positive blood CDST. Represents organisms introduced during blood sample collection or laboratory processing of the collected sample [18,44,45].

Admission to request for CDST: duration in days from the time of patient admission in the hospital to the time of blood CDST request in the patient’s EHR.

Request for CDST to receipt of blood sample at the laboratory: duration in days from time of test request logged in EHR to time of sample receipt at the laboratory.

Turnaround time (TAT): duration from specimen reception in the laboratory to the issuance of test report.

Admission to issuing of final blood CDST report: duration in days from time of admission to issuing of final test report. Used in this study as a proxy for lead time for blood CDST processing.

Lead-time for blood CDST processing of inpatients: duration from admission to targeted treatment.

### 2.6. Inclusion and Exclusion Criteria

Patients eligible for study inclusion had either a) at least one diagnosis of a primary or secondary BSI or b) a presumed BSI on their EHR. Blood CDST requests for outpatients were included only if the patient was subsequently admitted within seven days of specimen collection. Blood CDST of outpatients and of samples referred from other facilities were excluded.

### 2.7. Data Collection Process

Admission data and blood CDST performed for the study period were extracted from the EHR into Microsoft Excel and appended. Multiple patient and laboratory entries were merged based on the unique hospital ID, dates of admission, and culture request.

The entries were then filtered sequentially using the inclusion and exclusion criteria to generate the study population.

These entries were subsequently anonymised, assigned unique study identification numbers and exported for analysis.

### 2.8. Data Variables

Variables considered for all patients included the hospital ID, age, sex, dates of admission and discharge, ward of admission, clinical diagnosis and whether a blood CDST was requested. Variables for laboratory data extracted included the date of specimen receipt by the laboratory, the result of blood culture, whether a final report was issued, the date of the final report issue and whether clinical advice was given. Related variables for those with positive cultures were the bacterial isolates identified and the results of antimicrobial susceptibility testing for pathogens isolated.

### 2.9. Data Analysis

Analysis was performed using EpiData Analysis software (version 2.2.3.187, EpiData Association, Odense, Denmark) and Stata (version 16.0, Copyright 1985–2019, StataCorp LLC, College Station, TX, USA).

Categorical data were described using frequencies and percentages and presented in tables and figures. The numbers and proportions of patients with requests for blood CDST and undergoing blood CDST were calculated. Adjusted analysis using modified Poisson regression with robust variance estimator was conducted to assess the independent association of socio-demographic and clinical characteristics of the patients with request for blood CDST [46,47,48]. All the socio-demographic and clinical characteristics (age, gender, department, diagnosis and year) were included in the model. Prevalence ratios (PR) with 95% confidence interval (CI) were calculated as a measure of association. The number and proportion of patients with the request for blood CDST were also disaggregated by month to determine any trends over time. Key indicators of laboratory performance analysed included the diagnostic yield, the proportion of contaminations and the median (and inter-quartile range IQR) duration between the various stages from admission to the issuing of the final CDST reports were also determined. The antimicrobials tested for each isolate were compared with the CLSI Performance Standards for Antimicrobial Susceptibility Testing to determine suitability for the particular isolate. The dataset is deposited in figshare and is available under open share license CC BY 4.0 [49].

### 2.10. Ethics Considerations

Local ethics approval was granted by the Research Ethics Committee of the University of Health and Allied Sciences, Ho, with protocol number UHAS-REC A4 (1) 21–22. International ethical approval was additionally granted by the Ethics Advisory Group (EAG) of the International Union Against Tuberculosis and Lung Diseases with EAG number 42/21. The Research, Policy, Planning, Monitoring and Evaluation Directorate of HTH granted permission to access the EHR for the study.

Since this was a retrospective study using program data and did not directly recruit human participants, the issue of informed consent was waived. All data used from the general registry and laboratory database were extracted in a de-identified format (except the hospital identification number, which was essential for cleaning and validating the data. The hospital ID was removed after data cleaning and validation and was not used for analysis or presentation of results.

## 3. Results

### 3.1. Socio-Demographic and Clinical Characteristics

Infants (<1 year) constituted a third of inpatients with clinically diagnosed or suspected BSI. The median age of patients in this study was 28 (IQR: 0–56) years with a range of 0–105 years. Pneumonia was the most common 40.1% (1715/4278) diagnosis, followed by neonatal sepsis accounting for 22.2% (951/4278). The least common diagnosis was endocarditis 0.1% (3/4278). The mean hospital length of stay was 6.4 days (IQR: 2–7). Table 1 presents a summary of the socio-demographic and clinical characteristics of inpatients with BSI.

### 3.2. Blood Culture Requests by Clinicians

**Figure 1:**
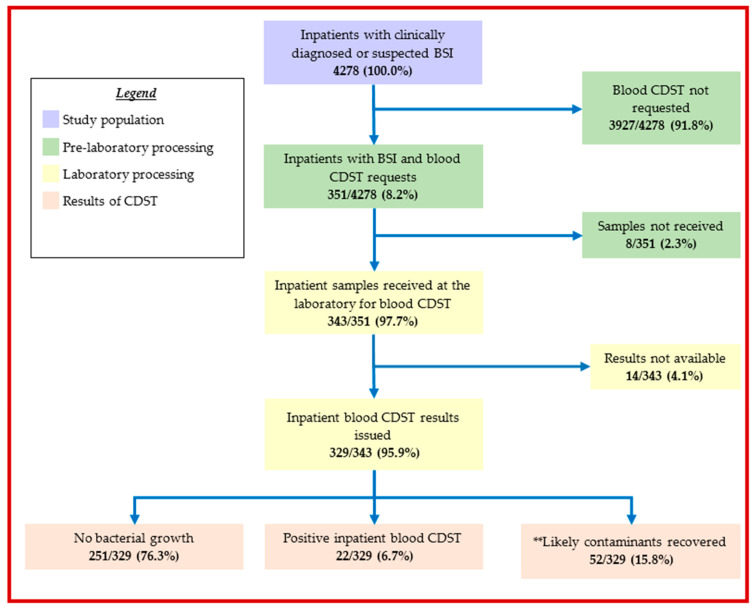
Blood culture use among inpatients with bloodstream infections at the Ho Teaching Hospital, Ghana: 2019–2021. ** Likely contaminants include: Coagulase-negative *Staphylococcus*, *Bacillus* and Diphtheroids; BSI = bloodstream infection; CDST = culture and drug susceptibility testing.

Of 4278 inpatients, 351 (8.2%) had blood CDST requested. Figure 1 summarises the sequence from admission to the issuing of blood CDST reports among inpatients during the study period.

**Figure 2:**
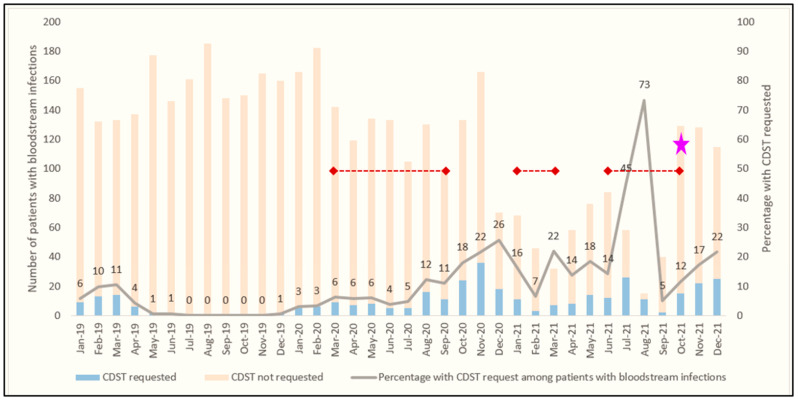
Trend in Blood CDST requests by month among inpatients with bloodstream infections at the Ho Teaching Hospital, Ghana: 2019–2021. ◆**--**◆ COVID-19 waves in Ghana; ★ New automated incubator operationalised.

Figure 2 shows the number of blood CDSTs performed by month in the study population. An average of three blood CDSTs was requested per month in 2019, 10 in 2020 and 22 in 2021 among inpatients with clinically diagnosed or suspected BSI.

### 3.3. Trends in Blood CDST Requests by Clinical Category

Table 1 shows the blood CDST requests for various inpatient categories. Of these, patients admitted to the Intensive Care Unit (PR—2.8, 95% CI: 1.6–4.8) were most likely to have had blood CDST requested for them. Additionally, patients with a diagnosis of sepsis other than neonatal sepsis (PR—3.3, 95% CI: 2.4–4.5) and those admitted in 2020 (PR—4.2; CI: 3.0–5.8) and 2021 (PR—6.3 CI: 4.5–8.9) were more likely to have received a blood CDST request.

### 3.4. Quality of Blood CDST Performed in Inpatients with Clinically Diagnosed or Presumed BSI

In over 99% of the blood CDST performed, only one sample was submitted per patient. There was only one patient for whom a pair of samples taken within 24 h was submitted for analysis. (Table 2).

**Figure 3:**
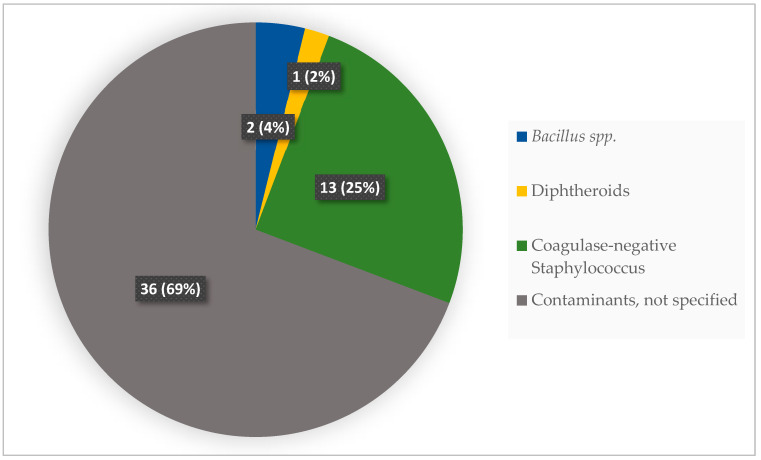
Contaminants isolated from blood CDST of inpatients of Ho Teaching Hospital, Ghana: 2019–2021. Values are presented as absolute numbers with percentages: N (%).

The total blood culture diagnostic yield was 6.7% (22/329) with contamination of 15.8% (52/329), as shown in Figure 1. Coagulase-negative *Staphylococcus* accounted for 25% (13/52) of contaminants isolated (Figure 3).

The majority of patients had blood CDST requested two days (IQR: 0–5) after admission, as shown in Table 3. All samples reached the laboratory on the same day (IQR: 0–0). The median TAT for laboratory processing (from the receipt of the blood sample at the laboratory to the issuing of the final blood CDST report) was 7 days (IQR: 5–9). The median lead time from admission to reporting of laboratory results was 10 days (IQR: 7–14).

A total of 22 bacterial pathogens were recovered from 329 blood samples. There were 97 instances in which the wrong antimicrobial was tested for the particular organisms and 159 instances in which a recommended antimicrobial for the organism was not tested (Supplementary Material: Table S1).

## 4. Discussion

This is the first study to examine blood CDST use based on clinical indication in Ghana. Our assessment established gaps in clinician requests for blood CDST and in laboratory procedures for processing blood CDST and recommends specific interventions aimed at improving clinical utility and laboratory quality of blood CDST and, consequently, treatment outcomes for patients admitted with BSI at HTH.

The age and sex distributions of the patients included in this study were similar to those of the population of the Volta region [29]. Considering all diagnoses, the Department of Obstetrics and Gynaecology bore the larger burden of inpatients [50]. However, when considering BSIs, Child Health and Accident and Emergency departments perceivably admitted more patients because they are more likely to cater to persons with sepsis or pneumonia, which were the two most common BSI diagnoses we found. The length of stay for persons with BSI was comparable to those of other acute care settings within the region [51].

Our major finding was the consistently low utilisation of blood CDST among clinicians managing inpatients with clinically diagnosed or suspected BSI admitted to HTH throughout the study period. This is at variance with national and international guidelines, which recommend blood CDST as part of the workup for each case of suspected BSI [2,17,26,39]. Even though persons admitted to the ICU or to the Department of Child Health were more likely to have had a request, blood CDST use in these departments was inadequate. A similarly low blood CDST utilisation (9%) was reported among inpatients in a referral hospital in Indonesia in 2017 [52]. However, reported utilisation among higher-income countries like Thailand, Israel and France in similar study populations is at least 21% [52,53,54], which could be due to a much better-developed health system.

Our finding confirms earlier reports of high use of empirical treatment in HTH and has dire consequences for the quality of care of such patients [27,28]. When blood CDST is used appropriately, it provides timely information for the clinical team to update empirical antimicrobial therapy, which is usually appropriately started at admission, converting it to targeted therapy earlier. This drives faster patient recovery, improves overall patient outcomes and minimises inappropriate use, which drives antimicrobial resistance. While the reasons for the low use are beyond the scope of our study, findings from other low resource settings that have recorded similarly low blood CDST utilisations have attributed them to factors such as physician attitudes, high costs, infrastructure and logistic challenges as well as the absence of quality assurance [13,19,20,55]. To better understand the reasons for low utilisation at HTH, qualitative studies are required to explore these reasons and to identify targeted areas for intervention, which will inform the development and implementation of clear local guidelines for the use of blood cultures within the hospital.

Our second most important finding was that even the few blood CDSTs performed had little clinical impact on patient care due to problems spanning the length of the laboratory diagnostic process from the pre-analytic procedures through the analytic and to the post-analytic phases. Sub-optimal blood sampling practices by the clinicians led to low diagnostic yield and high contamination of samples. At the laboratory end, long turnaround times, non-compliance to antimicrobial susceptibility testing guidelines and inadequate communication with the clinical teams led to delayed or inappropriate results. Understanding the reasons for these results in these parameters and all key quality indicators of blood CDST testing is an essential first step in improving the quality of the testing process.

The diagnostic yield of blood CDST describes the percentage recovery of pathogenic bacteria in BSI and is a critical parameter in any blood CDST quality assessment as it reflects true bacteraemia or fungaemia [40]. The expected diagnostic yield for blood CDST ranges from 10% to 15% [19,38,40] globally, with higher yields reported in the paediatric population [56]. The volume of blood obtained for each blood CDST request is critical for pathogen recovery in BSI, and these are clearly defined globally, specifying the number of blood samples to take and the amounts per sample bottle for both adult and paediatric patients [17,40]. Since, in our facility, unitary samples were taken for almost all blood CDST requests, the volumes per sample container could not be objectively measured as the in-house prepared sample collection bottle did not have fill volumes marked on them to guide the users.

Another factor that impacts diagnostic yield is the timing of sample collection. Contrary to international best practices, which recommend blood samples to be taken ideally within an hour of recognition of sepsis and prior to initiation of antimicrobials [2], we found a two-day lag between a clinical suspicion of BSI (including sepsis) and the issuance of a blood CDST request at HTH. During this period, empirical antimicrobial therapy is likely to have been initiated [27,28], further reducing the likelihood of pathogen recovery, especially in this facility, which employed in-house prepared culture media for most of the study duration. This problem is also compounded by the fact that being a tertiary facility, some of these patients would have received prior antimicrobial therapy either from their referring facilities or from self-administration as commonly practised in the community [57,58]. It is expected that with the installation and use of an automated blood culture incubation system in HTH, the issue of low yield attributable to prior antimicrobial therapy would be minimised as the commercially prepared culture media, which comes with the system, contains resins to bind antimicrobials present in the sample, thus improving diagnostic yield [38]. This system is additionally expected to improve pathogen recovery as the continuous monitoring of incubated samples for signs of growth further improves the sensitivity of the test [21,55]. The presence of facility-adapted guidelines coupled with continuous training would enhance proper utilisation and improve pathogen recovery and, therefore, yield blood CDST.

The second important quality indicator in blood CDST is the proportion of contaminants recovered, which ideally should not exceed 3% [17,45]. A recent study of blood cultures in another teaching hospital in Ghana revealed contamination of 3.6% among neonates and 13.9% among all other patients [12,59]. Another multi-centre study in Nigeria reported 7.8% contamination of blood cultures among both adult and paediatric patients [60]. Contamination in blood CDST denotes false positive results as it represents the proportion of organisms likely to have been introduced into the sample during sample collection or processing and, therefore, not likely to be responsible for the patient’s illness. High contamination rates prolong the process of pathogen identification and have implications for initiating targeted antimicrobial therapy. Furthermore, contamination of blood cultures leads to a waste of resources in the healthcare system.

The high proportion of contamination observed in this study implies an inadequacy of skin antisepsis prior to the procedure or inappropriate sampling, as most of the documented contaminants are known skin commensals. We also note that coagulase-negative *Staphylococcus,* the most common contaminant isolated from inpatient blood CDST during our study period, could be pathogenic in certain conditions. To assess this, the same organism must have been isolated from multiple blood CDST samples of the same patient. Another means of determining the pathogenic significance of a presumed contaminant is to analyse the time-to-detection of growth. Pathogenic bacteria are expected to grow faster than contaminants, and therefore, documenting the time-to-growth detection in blood CDST processing is an important variable in this determination [45]. However, none of these evaluations was possible as almost all the blood CDSTs performed were on unitary samples, and there were no timestamps in the HAMS software to facilitate this. Measures to minimise the risk of contamination of the blood cultures during sample collection include observing the five moments for hand hygiene by healthcare workers, preference of peripheral venipuncture oversampling through an existing cannula, adequate skin antisepsis, and the observation of aseptic technique for sampling preparation prior to venipuncture [17,61].

The TAT for laboratory processing of blood CDST samples is the third important measure of quality, specifically for the laboratory and generally for patient care [22,38]. As seen in our study, there was no delay in receiving samples at the laboratory after requests across all years. However, a major delay in the processing occurred at the laboratory analysis stage. This is an inherent feature of blood culture processing using phenotypic techniques, which are longer when compared to rapid molecular techniques [22]. A preliminary report for a positive blood CDST should be made immediately after the detection of growth. A negative CDST (no bacterial growth), on the other hand, requires at least 7 days of processing to report, making the standard TAT 3–8 days [19]. One reason for the median TAT of 7 days recorded in our study, therefore, could be the high level of negative samples reported. The measures discussed in the preceding paragraphs, which aim at improving the yield, would also shorten the time-to-positivity (time-to-growth detection) if implemented. Additionally, the introduction of the automated incubation system is expected to improve time-to-growth detection as it is designed to detect growth in real timecontinuous monitoring [62]. Other measures to improve TAT in general, including pre-analytical processes to enhance pathogen recovery pertaining to the timing of sample collection, the volume of draw and the number of samples, have already been discussed. It is expected that the results of blood CDST processing will be available to clinicians in time to direct the change from empirical to targeted therapy [19,43].

In this study, we used duration from admission to issuing of the laboratory report as a proxy for total lead time (duration from diagnosis to targeted treatment). In the median of 10 days it took for this process to occur within HTH, its clinical utility would likely have been lost [25] as most patients had exited the system (discharged, referred or dead) by the fourth day of admission. The two most important factors influencing this indicator were the duration from admission to clinical request and the laboratory TAT, which have already been discussed extensively. Other quality improvement measures for laboratory processing of blood CDST must focus on meticulous documentation of all laboratory processes and improving communication with the clinical team-in-charge, especially for preliminary reports, as these would help clinicians to modify their empirical treatments earlier [19,22]. Further studies to determine the treatment outcomes for these patients will be critical to quantifying the impact of these delays.

The last significant finding in our study, with major implications for quality assurance of laboratory processing for blood CDST samples, was the non-compliance to the CLSI guidelines (which the laboratory accedes to) for selecting antimicrobials for CDST of isolated pathogens [41]. Testing the wrong antimicrobial for an isolate could seriously undermine the role of the laboratory in determining targeted antimicrobial therapy, thus further limiting the clinical utility of the report generated. We surmise that a major reason for the high levels of non-compliance could be from logistical challenges faced at the laboratory. Testing of antimicrobials outside what is recommended must only be performed in consultation with the attending physician and the clinical pharmacy under the guidance of the antimicrobial stewardship team to be clinically useful.

Interventions for improvement focusing on pre-analytical factors, such as educating clinicians on the indications, timing, volume and number of blood CDST samples for enhanced diagnostic yield and reminders on aseptic techniques for sample collection, will help to improve the diagnostic yield and reduce contamination of blood CDST performed. Additionally, the laboratory must leverage the new automated blood culture incubator to improve the clinical utility by enhancing documentation of quality indicators like time-to-detection and instituting prompt and direct documentation and communication of preliminary results to the requesting clinician. Efforts must be made to strengthen the laboratory quality programme initiated using HTH, including a subscription to an external quality assurance programme. HTH can also leverage the use of the LHIMS EHR to improve the general quality of documentation, as this would be useful to conduct other operational research that will determine areas for quality improvement in other aspects of patient care.

### Strengths and Limitations

Strengths of this study include being the first to examine indications and quality of blood CDST in Ghana and following the STROBE (Strengthening the Reporting of Observational Studies in Epidemiology) guidelines for observational studies in our conduct and reporting [63]. Employing entire population sampling also enabled us to draw robust conclusions from the analysis. The choice of the study site is an additional strength as the HTH is a recently designated tertiary facility that is intent on improving the quality and scope of care in line with its new status and also had an EHR in place, which made data collection easier.

The first and major limitation of our study was missing data and variations in data capture among the two EHR systems, which posed challenges in the data analysis. Important laboratory processes like the results of Gram staining tests performed or issuance of preliminary CDST reports were not documented. Secondly, we were unable to assess the clinical significance of potential contaminants, such as coagulase-negative *Staphylococcus*, in at-risk individuals. Thirdly, the reasons for the low utilisation of CDST by the physicians could not be ascertained as that would require qualitative analysis, which was beyond the scope of this study. Fourthly, the small number of pathogens isolated made it impossible to draw any meaningful conclusions on the patterns of antimicrobial resistance found [64]. Finally, we were unable to assess the effect of the automated incubation system on blood CDST requests (if any) due to an insufficient number of time points to conduct a comprehensive time-series analysis, as it was operationalised three months to the end of the study.

Although we did not set out to determine the specific effect of the change in EHR software on blood CDST use, we opine that the decision to test is independent of the EHR system used because, in HTH, samples for blood CDST of inpatients are prioritised in laboratory processing and therefore, unlikely to be hindered by a delay in paperwork. Additionally, as the hospital was already using an EHR system, it was expected that the staff, being already conversant with one electronic system, would make an easier transition to a similar system in contrast to staff transitioning from a paper-based system to an electronic one.

## 5. Conclusions

We found low clinician requests for blood CDST for inpatients with suspected BSI. Furthermore, the clinical utility of the test for the few for whom it was requested was limited by a significant delay in the decision to request and some laboratory quality issues. These are important findings as the clinical relevance of blood CDST in managing patients with BSI and in AMR surveillance cannot be overemphasised. Further qualitative studies focusing on determining the hospital-specific reasons for underutilisation among clinicians in HTH would help to identify areas to further improve blood CDST services in the hospital. Efforts to improve antibiotic use at the HTH and other clinical care facilities will hinge on the initiation of a diagnostic stewardship programme, which involves improving culture utilisation as well as the production of quality-assured and timely CDST results for the management of patients. If the hospital and the nation are to make any gains in the emerging threat of AMR in Ghana, instituting a diagnostic stewardship programme at the facility and the national levels must be prioritised.

## Figures and Tables

**Table 1:** Socio-demographic and clinical characteristics of inpatients managed with bloodstream infections at the Ho Teaching Hospital, Ghana: 2019–2021.

Characteristics	Category Sub-Total * N (%)	Blood CDST ^#^ Requested n (%)	Adjusted PR ^^ (95% CI)
**Total**	4278 (100.0)	351 (8.2)	
**Age (years)**			
<1	1341 (31.4)	98 (7.3)	1
1–4	164 (3.8)	23 (14.0)	0.8 (0.5–1.3)
5–11	90 (2.1)	24 (26.7)	1.1 (0.7–1.8)
12–24	395 (9.2)	36 (9.1)	1.1 (0.5–2.6)
25–44	800 (18.7)	59 (7.4)	1.0 (0.4–2.3)
45–64	760 (17.8)	57 (7.5)	1.1 (0.5–2.6)
≥65	728 (17.0)	54 (7.4)	1.2 (0.5–2.8)
**Sex**			
Male	2103 (49.2)	185 (8.8)	1
Female	2175 (50.8)	166 (7.6)	0.8 (0.7–1.0)
Department **			
Child Health	1590 (37.2)	143 (9.0)	1.7 (0.7–4.0)
Accident and Emergency	1580 (36.9)	103 (6.5)	1
Internal Medicine	655 (15.3)	59 (9.0)	1.3 (1.0–1.8)
Obstetrics and Gynaecology	237 (5.5)	20 (8.4)	1.0 (0.6–1.7)
Surgery	186 (4.4)	12 (6.5)	**0.5 (0.3–0.9)**
Intensive Care Unit	30 (0.7)	14 (46.7)	**2.8 (1.6–4.8)**
Diagnosis ***			
Pneumonia	1715 (40.1)	72 (4.2)	1
Neonatal sepsis	951 (22.2)	48 (5.1)	1.1 (0.7–1.7)
Other Sepsis ^1^	679 (15.9)	91 (13.4)	**3.3 (2.4–4.5)**
Other ^2^	898 (21.0)	128 (14.3)	**3.9 (2.9–5.2)**
Missing	35 (0.8)	12 (34.3)	**11.0 (7.3–16.5)**
**Year**			
2019	1849 (43.2)	45 (2.4)	1
2020	1580 (36.9)	150 (9.5)	**4.2 (3.0–5.8)**
2021	849 (19.9)	156 (18.4)	**6.3 (4.5–8.9)**

* Column percentage with total as the denominator; ^#^ Row percentage with category subtotal as the denominator; ^^ Adjusted analysis using modified Poisson Regression; Statistically significant associations are shown in **bold**; CDST = culture and drug susceptibility testing; PR = prevalence ratio; ** Clinical department of ward first admitted. *** Only one diagnosis retrieved per patient; ^1^ Other Sepsis = septic shock, severe sepsis, sepsis, septicaemia, puerperal sepsis, septic abortion or sepsis following abortion; ^2^ Other = endocarditis, pyelonephritis, typhoid fever, typhoid perforation, osteomyelitis, septic arthritis, intra-abdominal or pelvic abscesses, bacterial peritonitis, cholecystitis and other inpatients with blood CDST requests.

**Table 2:** Number of blood CDST samples per set for inpatients of Ho Teaching Hospital: 2019–2021.

Samples Per Set	Number (%)
Solitary samples	309 (93.92)
Paired samples	2 (0.61)
Other multiple samples	18 (5.47)
Total	329 (100.00)

**Table 3:** Blood CDST processing times for inpatients: Ho Teaching Hospital, Ghana: 2019–2021.

Duration (Days)	Median	(IQR)
Admission to request for CDST (N = 351) *	2	(0–5)
Request for CDST to receipt of blood sample at the laboratory (N = 343) *	0	(0)
Receipt of blood sample at laboratory to issuing of final blood CDST report (N = 329) *^,#^	7	(5–9)
Admission to issuing of final blood CDST report (N = 329) *^,§^	10	(7–14)

* N, total number at each of the stages; CDST, culture and drug susceptibility testing; IQR, Interquartile range; ^#^ Laboratory turnaround time (TAT); ^§^ Lead time.

## Data Availability

The dataset is deposited in figshare with DOI: 10.6084/m9.figshare.20459835 and is available under an open share license CC BY 4.0.

## Supplementary Materials

The following supporting information can be downloaded at: https://www.mdpi.com/article/10.3390/ijerph20176631/s1, Table S1. Pathogens isolated and antimicrobial resistance patterns of inpatient blood CDST isolates: Ho Teaching Hospital, Ghana: 2019–2021.
