# Supplementary material for: Use and Quality of Blood Cultures for the Diagnosis of Bloodstream Infections: A Cross-Sectional Study in the Ho Teaching Hospital, Ghana, 2019–2021
Source: Int J Environ Res Public Health. 2023 Aug 23;20(17):6631. doi: 10.3390/ijerph20176631 (PMC10487590; doi:10.3390/ijerph20176631)
Supplement: Supplementary file 1 [file ijerph-20-06631-s001.zip › ijerph-2471824-supplementary.pdf]

Table S1: Pathogens isolated and antimicrobial resistance patterns of inpatient blood CDST isolates: Ho Teaching Hospital, Ghana: 2019-2021.

Organism	N	Antimicrobial resistance in isolate n/N																	MDR*
		Penicillins			Cephalosporins					Macrolides		Aminoglycosides		Quinolones		Others			
		amc	amp	pen	cxm	fox	ctx	ctr	caz	ery	van	gen	amk	cip	lvx	mem	cot	tet	
**Gram pos.**																			
S. aureus	2		1/2	1/1	0/1		0/1	0/1		0/1		0/2	0/1	0/2			1/1		0/2
S. pyogenes	1	1/1	0/1							0/1	0/1							0/1	0/1
**Sub-total**	**3**	**1/1**	**1/3**	**1/1**	**1/1**	**0/0**	**0/1**	**0/1**		**0/2**	**0/1**	**0/2**	**0/1**	**0/2**	**0/0**	**0/0**	**1/1**	**0/1**	**0/3**
**Gram neg.**																			
Klebsiella spp.	4		1/1	0/2	1/1		0/1	0/1	1/2		1/1	1/2	0/2	0/2	0/1		1/1	0/1	1/4
E. coli	3		3/3		3/3		2/3	2/3				1/2	0/3	0/1		3/3	2/3	1/1	3/3
Acinetobacter	2		1/1				0/1					1/2	0/2	0/2				2/2	0/2
Citrobacter	2		1/1		1/1		1/1	0/1					0/1			0/1	0/1		0/2
P. mirabilis	2			0/2														0/2	0/2
Pseudomonas	2											1/1	0/1	1/1	1/1	1/1			1/2
Salmonella sp.	2		0/1	0/1	1/1		1/1		1/1			1/2	0/1	1/2	0/1	1/2	0/1		1/2
Serratia	1					1/1		1/1	1/1			1/1		0/1	0/1	1/1			1/1
Francisella sp.	1		0/1		1/1					1/1		1/1		0/1		1/1	0/1	1/1	1/1
**Sub-total**	**19**	**0/0**	**6/8**	**0/3**	**7/7**	**1/1**	**4/7**	**3/6**	**3/4**	**1/1**	**1/1**	**7/11**	**0/10**	**2/10**	**1/4**	**7/9**	**3/7**	**4/7**	**8/19**

= recommended antimicrobials that were tested; = recommended antimicrobials that were not tested; = antimicrobials that were not recommended for the particular organism; = MDR strains; *MDR= Multi-drug resistance (resistance to three or more classes of antimicrobials); amc= amoxicillin+clavulanate; amp= ampicillin; pen= penicillin; cxm= cefuroxime; fox= cefoxitin; ctx= cefotaxime; ctr= ceftriaxone; caz= ceftazidime; ery= erythromycin; van= vancomycin; gen= gentamicin; amk= amikacin; cip= ciprofloxacin; lvx= levofloxacin; mem= meropenem; cot= sulfamethoxazole+trimethoprim; tet= tetracycline; chl= chloramphenicol.
